# Design, comparison and selection of two competition incentive mechanisms

**DOI:** 10.1371/journal.pone.0345083

**Published:** 2026-04-01

**Authors:** Dahai Li, Huan Wang, Jingcheng Ye

**Affiliations:** 1 School of Big Data and Statistics, Anhui University, Hefei, Anhui Province, China; 2 School of Management, Hefei University of Technology, Hefei, Anhui Province, China; 3 School of Finance, Shanghai University of Finance and Economics, Shanghai, China; 4 Wu Jinglian School of Economics, Changzhou University, Changzhou, Jiangsu Province, China; 5 Hengyang Normal University, Facuity of Economics and Management, Hengyang, Hunan Province, China; Universitat Jaume I, SPAIN

## Abstract

When facing multiple agent teams, principals often implement competitive mechanisms to mitigate moral hazard. These mechanisms can be categorized as either result-oriented or process-oriented, depending on their operational focus. This paper analyzes the optimal incentive contracts offered by principals to agents under these two competitive modes. Furthermore, to mitigate potential conflicts arising from these mechanisms, the matching of agents with varying abilities is also considered. Our analysis reveals that both competitive modes can effectively incentivize agent effort. However, excessive result-oriented competition can be detrimental to the principal’s benefits, suggesting that its intensity should be carefully calibrated. Moreover, when forming agent pairs, a “strong-weak” pairing is preferable to a “strong-strong” pairing, which, in turn, is preferable to a “weak-weak” pairing. Finally, this paper discusses the scope of application for each competition-incentive system. These findings provide a foundation for effectively utilizing competitive mechanisms to enhance the principal’s returns.

## 1 Introduction

The engagement of multiple agents by a principal gives rise to moral hazard problems. Even in the absence of risk in the production or sales process, agents may shirk their responsibilities and attribute blame to one another, resulting in suboptimal effort levels [1]. While incentive mechanisms are essential, they are not always sufficient to fully address this challenge. The literature suggests that principals can further enhance productive efficiency by implementing competitive mechanisms that incentivize agents to exert greater effort [2,3]. These competitive mechanisms are broadly categorized into two types: endogenous process-oriented competition, where agents’ production or sales activities are interdependent; and result-oriented competition, where agents operate independently, but their individual performance is subject to comparative evaluation [4,5].

Consider a manufacturer, denoted as *P*, who produces a certain product and engages multiple retailers, denoted as $A_{1},A_{2},\ldots ,A_{N}$, as sales agents. The manufacturer, *P*, can stimulate competition among these retailers through two distinct strategies. The first strategy involves placing at least two retailers within the same city, thereby inducing competition for market share. Consequently, an increase in one retailer’s market share necessarily entails a decrease in the market share of others. The second strategy involves situating retailers in geographically distinct markets (different cities), minimizing direct competition for market share. However, the manufacturer rewards retailers based on their relative sales performance (sales volume rankings), penalizing those with lower rankings. In the first strategy, the retailers’ sales processes are interdependent; the efforts of one sales team not only enhance profits within their existing market share but also enable the capture of market share from competing teams. This constitutes an endogenous, process-oriented competitive model. Conversely, in the second strategy, due to the absence of market overlap, the manufacturer, as the principal, focuses solely on annual sales volume to determine rewards and penalties. This represents an exogenous, result-oriented (or outcome-focused) competition scheme.

In fact, neither of the two schemes is perfect. Under the first strategy, agents may expend considerable efforts in vying for market share, potentially leading to wasteful duplication of efforts. This necessitates addressing the assortative matching problem [6], which concerns the optimal pairing of agents with varying competencies to maximize the principal’s returns. This paper demonstrates that a “strong-weak” pairing is optimal, followed by a “strong-strong” pairing, with a “weak-weak” pairing being the least effective. This finding is consistent with the principle of negative assortative matching. Under the second strategy, the principal’s ranking based on final performance introduces additional competitive risk for agents, which is exacerbated by artificially intensified competition. Furthermore, the appropriate level of competition intensity must be determined for retailers in different cities under this strategy. This paper demonstrates that the optimal competition intensity is correlated with agents’ individual efficiency; more efficient agents require stronger competitive incentives. This implies that establishing uniform competition across agents with heterogeneous efficiencies is suboptimal, and that indefinitely increasing competition intensity ultimately diminishes the principal’s returns.

Early research on multi-agent problems has highlighted the importance of structuring agents into teams. Agents, when organized into teams, can collectively contribute to a firm’s production and sales activities; moreover, with increasing team size, economies of scale in information processing can be realized [7]. By consolidating previously disparate transactions within a single firm, the principal can more effectively generate value through information utilization. However, the potential for internal collusion among agents [8] or mutual shirking can exacerbate moral hazard [1].

Competitive mechanisms offer a potential avenue for mitigating moral hazard problems arising in multi-agent settings. Holmstrom [1] indicates that outcome-based competition among agents can effectively alleviate moral hazard. Bridgman [2] notes that competition enhances output efficiency, and Dulleck et al. [3] observes that in asymmetric service markets between buyers and sellers, competition significantly increases market transactions. While competition encourages agents to exert effort, it does not necessarily guarantee increased profits for the principal. Rud et al. [9] and Li et al. [10] point out that excessive competition can exacerbate moral hazard, thereby harming the principal’s returns. Overcompetition can also negatively impact market equilibrium. Bolton et al. [11] note that excessive competition in the rating agency market reduces market efficiency, as sellers will only pay for favorable ratings. It is noteworthy that through the establishment of a principal-agent model, this paper also demonstrates that excessive competition can reduce the principal’s profits. In terms of model setting, Li et al. [12] posit that when an agent’s effort level increases, they can not only expand their own market share but also reduce the market share of other agents. Therefore, the effort processes between agents influence each other and generate competition. This competition closely resembles the output competition in Cournot and Stackelberg models, except that the independent variable is the agent’s effort rather than output. This process-oriented competitive model can effectively address moral hazard issues caused by information asymmetry. In contrast to process competition, Holmstrom [1] and Xu [4] argue that competition within a team should rely solely on output outcomes as the reference variable, and the optimal contract within a team depends only on the individual final output of the agent and the average output outcome of multiple agents. Therefore, this paper examines incentive contracts under both process-oriented and result-oriented competitive models and compares the advantages and disadvantages of the two competitive systems through comparative static analysis.

In this study, our objective is to construct a simple model to study the relationship between competition and incentives within a single period [0,*T*]. In practice, due to legal constraints and menu costs, incentive contracts and competitive arrangements are indeed fixed for a certain duration [0,*T*]. When solving the model, we deliberately depart from the martingale method and instead adopt the certainty-equivalent approach, because our research setting is not one of repeated games. According to reference [13], the martingale method proposed by Sannikov is an approach for analysing repeated games [14]. The core of the martingale method is to characterize the optimal contract using the state variable continuation value. This fundamental approach was first proven to be effective by Spear and Srivastava in the study of infinitely repeated games [15], and Sannikov utilizes this conclusion. Specifically, we analyze competition and incentives in a finite-horizon, non-repeated game framework, following the methodology of Holmstrom and Milgrom [16], who also employed the certainty-equivalent method in their “Brownian model.” Under the Brownian motion framework with CARA preferences and linear contracts, Holmstrom and Milgrom (Theorem 7) show that the optimal solution to the principal’s problem is to instruct the agent to choose a deterministic effort level, which is independent of the realization history of the stochastic shocks. This key insight is also clearly summarized in Acemoglu and Autor’s Lectures in Labor Economics (p. 90) [17].

We adopt the model framework of Holmstrom and Milgrom [16], incorporating a temporal dimension to derive the optimal competition-incentive contract in continuous time. Our model incorporates Brownian motion into the production function, which exhibits increasing variance with time, implying that longer contract durations are associated with greater output risk. According to standard principal-agent theory, agent incentives typically decrease with increasing risk [18]. This might lead one to conclude that longer contract durations should result in weaker incentives. However, this conclusion contradicts observed practice, where employers often offer greater incentives to retain employees for longer periods. This paper resolves this apparent paradox by constructing a continuous-time principal-agent model demonstrating that optimal incentives are positively correlated with contract duration under both strategies. Furthermore, both competition-incentive schemes effectively incentivize greater effort compared to a scenario without competition. Finally, we explore the relative applicability of the two schemes, concluding that result-oriented competition is more advantageous when there are substantial disparities in agent incentives.

In summary, existing research on competition-incentive contracts within finite-horizon, non-repeated game settings is limited, particularly concerning comparative analyses of process-oriented and result-oriented competition mechanisms. Therefore, this paper develops a principal-agent model to specifically investigate the following questions: (1) how to design the optimal contract under process-oriented competition; (2) how to design the optimal contract under result-oriented competition; and (3) a comparative static analysis of process-oriented versus result-oriented competition. The primary contributions of this paper are threefold: first, we introduce competition mechanisms into traditional incentive contracts by proposing and constructing a “competition-incentive” model; second, we examine the relationship between continuous-time incentive contract design and competition within a non-repeated game framework; and third, we analyze both process-oriented and result-oriented competition models, discussing the relative applicability of these two competition-incentive systems.

The organization of this paper is as follows: Section 2 provides a description and the assumptions of the model. Section 3 solves and analyzes the constructed model. Section 4 conducts a comparative static analysis of the two “competition-incentive” mechanisms using numerical simulation methods. The final section summarizes the paper. All proofs are presented in the Appendix.

## 2 Problem description and model assumptions

**Fig 1:**
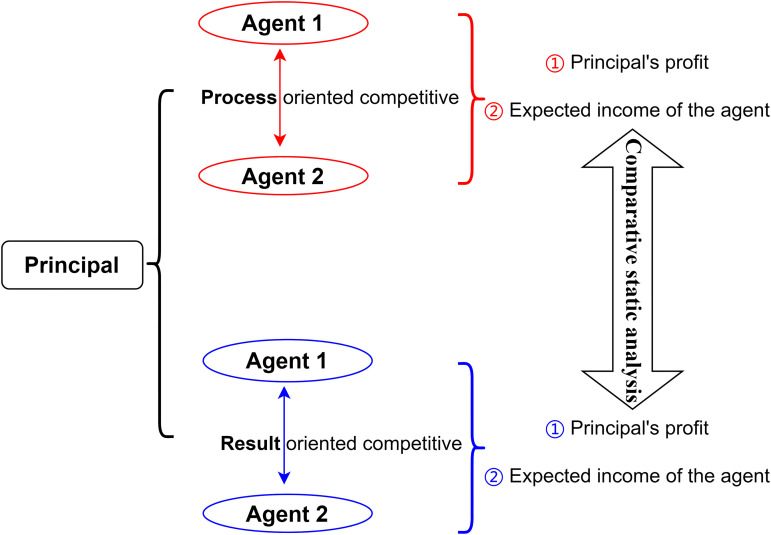
Diagram of comparative static analysis based on two competitive models.

Consider a sales market where a principal (manufacturer) engages two agents (retailers) to sell products. To incentivize agent effort, the principal implements a competitive mechanism. One common approach is to compare sales performance [1,4,5] and reward or penalize agents accordingly, thereby establishing a competitive mechanism. Alternatively, as discussed in [10], the principal can position the two agents within the same market. Given the finite market capacity, their sales activities will inherently be competitive; an agent’s earnings depend not only on their own effort level but also on the effort exerted by the other agent. Holding the opponent’s effort constant, increasing one’s own effort can attract more customers. This paper employs a model to conduct a comparative static analysis of these two competitive modes. Fig 1 presents a basic framework diagram of this paper. For convenience, we begin with the following assumptions.

**Assumption 1** Following Holmstrom’s framework [16], we assume that the principal, *P*, is risk-neutral, while the agent, *A*_*i*_, is risk-averse. The agent’s utility function is given by U=−exp(−ρΠ), where ρ denotes the Arrow-Pratt coefficient of absolute risk aversion and Π represents the agent’s earnings.

**Assumption 2** The principal and the agent have information asymmetry, where the principal cannot observe the agent’s level of effort but can only observe the value process of sales or output *X*(*t*), and sets the incentive contract based on this observable performance.

**Assumption 3** If the agents operate independently, the value process generated by agent *A*_*i*_ satisfies $dX_{i}(t)=k_{i}a_{i}(t)dt+\sigma_{i}dW_{t}^{i}$ [14]. Here, *k*_*i*_ is the efficiency of the agent *A*_*i*_’s effort; *a*_*i*_(*t*) denotes the effort process; $W_{t}^{i}$ is a s*t*andard Brownian motion, used to characterize the randomness of the value process; $\sigma_{i}$ is the volatility of the value process and i∈{1,2}.

**Assumption 4** The cost incurred by agent *A*_*i*_ at time *t* for exerting effort *a*_*i*_(*t*) is given by $C(a_{i})=c_{i}{a_{i}}^{2}(t)/2$ [19,20]. A smaller value of *c*_*i*_ indicates that the agent is more efficient.

All model parameters and decision variables are provided in Table 1 for reference. The efficiency parameters $k_{1},k_{2}$ determine how effectively agents’ efforts are translated into outputs. The parameter range of $k_{1},k_{2}$ is (0,+∞). The competition intensity parameter *J*_*i*_ captures the strength of rivalry between agents. Higher *J*_*i*_ reflects more intense competition, the range of *J*_*i*_ is [0,1]. The risk-aversion coefficient ρ measures the agent’s degree of absolute risk aversion; a larger ρ implies stronger risk aversion, the range of ρ is [0,+∞]. The cost parameter *c*_*i*_ represents the marginal cost of efforts. Larger *c*_*i*_ corresponds to higher effort costs for the agent, the range of *c*_*i*_ is [0,+∞]. The volatility parameter $\sigma_{i}$ characterizes the randomness of the project’s output; larger $\sigma_{i}$ indicates greater uncertainty, the range of $\sigma_{i}$ is [0,+∞]. The contract duration is defined over the finite interval [0,*T*], and therefore requires *T* > 0. These clarifications give a better understanding about the economic meaning and feasible ranges of the parameters used in our numerical exercises.

**Table 1:** Description of parameters.

Symbols	Description	Symbols	Description
*P*	Principal	*X*_*i*_(*t*)	The value process generated by agent *A*_*i*_
*A*	Agent	$\sigma_{i}$	The volatility of the value process *X*_*i*_(*t*)
ρ	Absolute risk aversion coefficient	*k*_*i*_	The production efficiency of agent *A*_*i*_
C(·)	Effort cost function	*W*_*i*_(*t*)	The standard Brownian motion associated with *X*_*i*_(*t*)
*c*_*i*_	The cost factor of agent *A*_*i*_	$\Pi_{i}$	The payoff of agent *A*_*i*_
$\alpha_{i}(t)$	The fixed wage of agent *A*_*i*_	$\beta_{i}$	The incentive of agent *A*_*i*_
*a*_*i*_(*t*)	The effort process of agent *A*_*i*_	*T*	The term of the contract is [0, T]
${\bar{U}}_{i}$	The reservation utility of agent *A*_*i*_	*J*_*i*_	The competition intensity of agent *A*_*i*_

## 3 Model construction and solution

### 3.1 Process-oriented competitive mode

Process competition typically arises among agents operating within the same market. Following Hoppe & Kusterer and Duan et al. [21,22], process-oriented competition can be interpreted as an externality of effort. For example, consider a principal *P*, a manufacturing company, who engages two retailers, *A*_1_ and *A*_2_, as sales agents. If retailer *A*_1_ exerts greater effort and captures a larger market share, this will necessarily reduce the sales volume of retailer *A*_2_. Alternatively, consider a scenario where the principal requires a batch of products and engages two agents, *A*_1_ and *A*_2_, to handle research and development as well as production. Increased effort by agent *A*_1_ will inevitably affect the principal’s procurement volume from agent *A*_2_. In both cases, a process-oriented competitive dynamic exists between agents *A*_1_ and *A*_2_.

The principal establishes the contract duration as [0, *T*]. Following Holmstrom [16], we assume that contracts within the interval [0, *T*] remain static. In the absence of competition, the utility obtained by agent *A*_*i*_ is given by:


$$\Pi_{A_{i}}(T)=\beta_{i}\left[\int_{0}^{T}e^{-rt}k_{i}a_{i}(t)dt+\int_{0}^{T}e^{-rt}\sigma_{i}dW_{t}^{i}\right]+\int_{0}^{T}e^{-rt}\alpha_{i}(t)dt-\int_{0}^{T}e^{-rt}\frac{c_{i}}{2}{a_{i}}^{2}(t)dt.$$ (1)

where $\beta_{i}$ is the incentive factor in the contract, $\int_{0}^{T}e^{-rt}k_{i}a_{i}(t)dt$ represents the present value of output generated through effort, $\int_{0}^{T}e^{-rt}\sigma_{i}dW_{t}^{i}$ represents the present value of risk associated with the sales process, $\int_{0}^{T}e^{-rt}\alpha_{i}(t)dt$ represents the present value of the fixed salary, and $\int_{0}^{T}\frac{e^{-rt}c_{i}}{2}a_{i}^{2}(t)dt$ represents the present value of the cost of effort.

Due to the presence of competition, agent *A*_*i*_ experiences a gain or loss in utility as a result of the competitive process. The post-competition utility function is given by:


$$\begin{array}{cc} {\tilde{\Pi }}_{A_{i}}(T) & =\beta_{i}\left[\int_{0}^{T}e^{-rt}k_{i}a_{i}(t)dt+\int_{0}^{T}e^{-rt}\sigma_{i}dW_{t}^{i}+J_{i}\int_{0}^{T}e^{-rt}[a_{i}(t)-a_{-i}(t)]dt\right]\\ & +\int_{0}^{T}e^{-rt}\alpha_{i}(t)dt-\int_{0}^{T}e^{-rt}\frac{c_{i}}{2}{a_{i}}^{2}(t)dt. \end{array}$$

The impact of competition on profit $\Pi_{A_{i}}(T)$ is represented by $J_{i}\int_{0}^{T}e^{-rt}[a_{i}(t)-a_{-i}(t)]dt$, where agent *A*_*i*_ increases effort to capture market share that would have otherwise belonged to agent $A_{-i}$. The parameter *J*_*i*_ represents the competition intensity.

Given that the agent’s utility function is of the absolute risk aversion form. According to the Ito Isometry property, the certainty equivalent of its utility function can be derived.


$$\begin{array}{cc} {\tilde{U}}_{A_{i}}(T) & =\beta_{i}\left[\int_{0}^{T}e^{-rt}k_{i}a_{i}(t)dt+J_{i}\int_{0}^{T}e^{-rt}[a_{i}(t)-a_{-i}(t)]dt\right]+\int_{0}^{T}e^{-rt}\alpha_{i}(t)dt\\ & -\int_{0}^{T}\frac{c_{i}e^{-rt}}{2}{a_{i}}^{2}(t)dt-\frac{\rho_{i}}{2}\beta_{i}^{2}\sigma_{i}^{2}\int_{0}^{T}e^{-2rt}dt. \end{array}$$ (2)

In this context, the expected present value of total revenue derived from agent *A*_*i*_’s individual effort and the competition inherent in the contract is given by$\int_{0}^{T}e^{-rt}k_{i}a_{i}(t)dt+J_{i}\int_{0}^{T}e^{-rt}[a_{i}(t)-a_{-i}(t)]dt.$ The principal allocates a fraction $\beta_{i}$ of this revenue to the agent as an incentive. Given the inherent risk in the value process and the agent’s risk aversion, a risk premium is incorporated, which is given by $\frac{\rho }{2}\beta_{i}^{2}\sigma_{i}^{2}\int_{0}^{T}e^{-2rt}dt.$

The payoff that the principal *P* receives is:


$${\tilde{\Pi }}_{P}(T)=\sum_{i}(1-\beta_{i})\left\{\int_{0}^{T}e^{-rt}k_{i}a_{i}(t)dt+J_{i}\int_{0}^{T}e^{-rt}[a_{i}(t)-a_{-i}(t)]dt\right\}-\int_{0}^{T}e^{-rt}\alpha_{i}(t)dt.$$ (3)

After allocating a fraction $\beta_{i}$ of the revenue to agent *A*_*i*_, the principal, *P*, retains the remaining $\left(1-\beta_{i}\right)$. Furthermore, the principal must cover the present value of fixed wages for both agents, given by $\sum_{i}\int_{0}^{T}e^{-rt}a_{i}(t)dt$. As the principal is risk-neutral, she do not incur any risk premium cost.

This paper assumes that agents are rational, who seek to maximize their individual utility and thus must satisfy the incentive compatibility (IC) constraint. Furthermore, the utility obtained by agent *A*_*i*_ is no less than their reservation utility, $\bar{U}$, satisfying the individual rationality (IR) constraint. Consequently, we formulate the following Model I:


$$\begin{array}{c} \max{\tilde{\Pi }}_{p}(T)\;\text{ s.t. }\left\{ \begin{array}{cc} \left(IR\right) & {\tilde{U}}_{A_{i}}(T)\geq {\bar{U}}_{i}\\ \left(IC\right) & a_{i}(t)\in \arg\max{\tilde{U}}_{A_{i}}(T) \end{array}\right.\; \end{array}$$

The following conclusion can be drawn from solving Model I.

**Propositon 1.** Under the process-oriented competition model, the optimal incentive in the contract is given by:


$${\tilde{\beta }}_{i}(T)=\frac{2{\left(k_{i}+J_{i}\right)}^{2}-2J_{-i}(k_{i}+J_{i})}{2{\left(k_{i}+J_{i}\right)}^{2}+\rho_{i}\sigma_{i}^{2}c_{i}(1+e^{-rT})}.$$

**Proof.** The proof of Proposition 1 is provided in Appendix.

Proposition 1 provides the expression for the optimal incentive under process-oriented competition. To ensure the incentive $\tilde{\beta }>0$, the condition $k_{i}+J_{i}>J_{-i}$ must hold. Further analysis of $\tilde{\beta }$ leads to the following corollary.

**Corollary 1.** Under the process-oriented competition mechanism, the contractual incentive for agent *A*_*i*_ increases with the contract duration *T*, their own efficiency *k*_*i*_, and the competition intensity *J*_*i*_, while it decreases with the opposing agent’s competition intensity $J_{-i}$.

In the classical principal-agent model, agents’ incentives depend solely on their own efficiency. When engaging a more capable agent, the principal must offer greater incentives [18], a conclusion encompassed by Corollary 1. However, in a competitive environment, agent *A*_*i*_’s incentives are also influenced by competition from rivals. An increase in the competitor’s intensity, $J_{-i}$, reduces the incentives offered to *A*_*i*_ by the principal. Finally, a longer contract duration, *T*, necessitates greater incentives from the principal to maintain the principal-agent relationship.

As production or sales progress, the associated risks increase over time. According to standard principal-agent theory [18], the incentives offered to a risk-averse agent typically decrease with increasing risk, suggesting that the incentive ${\tilde{\beta }}_{i}(T)$ should decrease as *T* increases. However, this contradicts observed practice, primarily because standard principal-agent models do not account for the time discounting of risk. In Model I, we consider not only the increasing effect of risk but also the fact that the present value of risk-related gains (or losses) diminishes over time. This explains why the principal must offer greater incentives to the agent as the contract duration, *T*, extends. This phenomenon is commonly observed. For example, in China, universities typically offer more attractive compensation packages for faculty positions with longer service requirements.

**Corollary 2.** Under the process-oriented competition model, agents are motivated to exert more effort. The effort exerted by agent *A*_*i*_ is


$${\tilde{a}}_{i}(T)=\frac{2{\left(k_{i}+J_{i}\right)}^{3}-2J_{-i}{\left(k_{i}+J_{i}\right)}^{2}}{2c_{i}{\left(k_{i}+J_{i}\right)}^{2}+\rho_{i}\sigma_{i}^{2}c_{i}^{2}(1+e^{-rT})}.$$

Further analysis of Corollary 2 yields $\partial {\tilde{a}}_{i}(T)/\partial J_{i}>0$, which confirms the conclusion that competition motivates agents to exert more effort, thereby providing indirect validation of the fact that higher competition intensity is associated with a greater willingness to work hard. Additionally, it is shown that $\partial {\tilde{a}}_{i}(T)/\partial T>0$, indicating that the effort exerted by the agent is positively correlated with the length of the contract term. This aligns with real-world practices, where companies are indeed more inclined to offer long-term contracts to hardworking employees.

Corollary 2 also reveals that $\partial {\tilde{a}}_{i}(T)/\partial J_{-i}<0$, indicating that an increase in the competition intensity of other agents reduces the effort level of agent *A*_*i*_. This highlights the importance of strategically pairing agents with varying competition intensities. Based on Corollary 2, it can be inferred that a “strong-strong” pairing is not necessarily superior to a “strong-weak” pairing. We further investigate the agent pairing problem in Section 4.

### 3.2 Result-oriented competition model

The preceding analysis focus on process-oriented competition, where the competitive mechanism involves agents vying for each other’s resources and market share through continuous efforts, thereby increasing their own returns. However, another prevalent competition model uses outcomes as the sole performance metric. Consider, for instance, a manufacturer facing a large market who assigns one agent to manage sales in city A and another in city B. Due to geographical separation, there is no direct operational interaction between the two sales agents, and their efforts do not directly compete. Nevertheless, to further incentivize agent effort, the manufacturer establishes a result-oriented competition by comparing their respective sales performances.

Define the competitive outcome between agent *A*_*i*_ and the other agent as


$$\Delta_{i}=\int_{0}^{T}e^{-rt}k_{i}a_{i}(t)dt+\int_{0}^{T}e^{-rt}\sigma_{i}dW_{t}^{i}-\left[\int_{0}^{T}e^{-rt}k_{-i}a_{-i}(t)dt+\int_{0}^{T}e^{-rt}\sigma_{-i}dW_{t}^{-i}\right].$$

Here, $\int_{0}^{T}e^{-rt}k_{i}a_{i}(t)dt+\int_{0}^{T}e^{-rt}\sigma_{i}dW_{t}^{i}$ represents the final outcome resulting from the effort exerted by agent *A*_*i*_. By comparing this outcome with that of the other agent, we establish the criterion for result-oriented competition, denoted as $\Delta_{i}$. Applying the properties of Ito’s integral, we obtain


$$E(\Delta_{i})=\int_{0}^{T}e^{-rt}k_{i}a_{i}(t)-e^{-rt}k_{-i}a_{-i}(t)dt;\;Var(\Delta_{i})=\frac{\left(\sigma_{1}^{2}+\sigma_{2}^{2})(1-e^{-2rT}\right)}{2r}.$$

In a result-oriented competitive environment, the final utility of agent *A*_*i*_ is


$$\begin{array}{cc} {\hat{\Pi }}_{A_{i}}(T) & =\beta_{i}\left[\int_{0}^{T}e^{-rt}k_{i}a_{i}(t)dt+\int_{0}^{T}e^{-rt}\sigma_{i}dW_{t}^{i}\right]+e^{-rT}J_{i}\Delta_{i}\\ & +\int_{0}^{T}e^{-rt}\alpha_{i}(t)dt-\int_{0}^{T}\frac{e^{-rt}c_{i}}{2}{a_{i}}^{2}(t)dt. \end{array}$$ (4)

Here, $\beta_{i}\left[\int_{0}^{T}e^{-rt}k_{i}a_{i}(t)\;dt+\int_{0}^{T}e^{-rt}\sigma_{i}\;dW_{t}^{i}\right]$ represents the revenue obtained by agent *A*_*i*_ through their own effort; $J_{i}\Delta_{i}$ represents the impact of the competitive outcome on their final utility. As this competition model focuses solely on outcomes, the competitive rewards (or penalties) are realized at the terminal time *T*, hence the discount factor is $e^{-rT}$. Additionally, over the time interval [0, *T*], the present value of the fixed wage received by the agent is given by $\int_{0}^{T}e^{-rt}\alpha_{i}(t)\;dt$, and the present value of the cost of effort is given by $\frac{1}{2}\int_{0}^{T}e^{-rt}c_{i}a_{i}^{2}(t)\;dt.$

According to the Ito Isometry property, the certainty equivalence for agent *A*_*i*_ under a result-oriented competition can be derived as


$$\begin{array}{cc} {\hat{U}}_{A_{i}}(T) & =\beta_{i}(T)\int_{0}^{T}e^{-rt}k_{i}a_{i}(t)dt+e^{-rT}J_{i}E(\Delta_{i})+\int_{0}^{T}e^{-rt}\alpha_{i}(t)dt\\ & -\int_{0}^{T}\frac{e^{-rt}c_{i}}{2}{a_{i}}^{2}(t)dt-\frac{\rho_{i}}{2}\left[\frac{\beta_{i}^{2}(T)\sigma_{i}^{2}(1-e^{-2rT})}{2r}+e^{-2rT}J_{i}^{2}var(\Delta_{i})\right]. \end{array}$$ (5)

Here, $\beta_{i}(T)\int_{0}^{T}e^{-rt}k_{i}a_{i}(t)\;dt$ represents the return on the effort exerted by agent *A*_*i*_, and $e^{-rT}J_{i}E(\Delta_{i})$ represents the expected reward (or penalty) due to competition based on final performances. Additionally, over the time interval [0, *T*], the present value of the fixed wage received by the agent is given by $\int_{0}^{T}e^{-rt}\alpha_{i}(t)\;dt$, and the present value of the effort cost is $\frac{1}{2}\int_{0}^{T}e^{-rt}c_{i}a_{i}^{2}(t)\;dt.$ Due to the incomplete controllability of the valuation process, a risk premium is incurred, given by $\frac{\rho_{i}}{2}\cdot \frac{\beta_{i}^{2}(T)\sigma_{i}^{2}(1-e^{-2rT})}{2r}.$ Furthermore, in result-oriented competition, the two agents’ final outcomes are compared, with $\Delta_{i}$ serving as the basis for this comparison. As $\Delta_{i}$ is a random variable, it introduces additional competitive risk. The resulting risk premium is given by $\frac{\rho_{i}}{2}\cdot e^{-2rT}J_{i}^{2}\text{Var}(\Delta_{i}).$

The principal’s utility is


$${\hat{\Pi }}_{P}(T)=\sum_{i}\left(1-\beta_{i})\int_{0}^{T}e^{-rt}k_{i}a_{i}(t)dt-\int_{0}^{T}e^{-rt}\alpha_{i}(t)dt-e^{-rT}J_{i}E(\Delta_{i}\right).$$ (6)

The principal’s revenue is given by $(1-\beta_{i})\int_{0}^{T}e^{-rt}k_{i}a_{i}(t)\;dt.$ The principal also incurs the cost of the agent’s fixed wage, given by $\int_{0}^{T}e^{-rt}\alpha_{i}(t)\;dt.$ The term $e^{-rT}J_{i}E(\Delta_{i})$ represents the expected reward (or penalty) paid by the principal to the winning (or losing) agent in the result-oriented competition. Therefore, we formulate the following principal-agent Model II.


$$\begin{array}{c} \max_{\alpha ,\beta ,T}{\hat{\Pi }}_{P}(T)\;\text{ s.t. }\left\{ \begin{array}{cc} \left(IR\right) & {\hat{U}}_{A_{i}}(T)\geq {\bar{U}}_{i}\\ \left(IC\right) & a_{i}(t)\in \arg\max{\hat{U}}_{A_{i}}(T) \end{array}\right.\; \end{array}$$

Substituting equations (5) and (6) into Model II allows us to obtain the following conclusions.

**Proposition 2.** Under the result-oriented competition model, the optimal incentive in the contract is given by


$${\hat{\beta }}_{i}(T)=\frac{2k_{i}^{2}(1-e^{-rT}J_{i})}{2k_{i}^{2}+c_{i}\rho_{i}\sigma_{i}^{2}(1+e^{-rT})}.$$

**Proof.** The proof of Proposition 2 is provided in Appendix.

Proposition 2 provides the expression for the optimal incentive under result-oriented competition, and further analysis of ${\hat{\beta }}_{i}(T)$ leads to the following corollary.

**Corollary 3.** The result-competition factor *J*_*i*_ mitigates the incentive, but the duration *T* and efficiency *K*_*i*_ enhance the incentive.

Comparing with Corollary 1, we observe that in a result-oriented competitive environment, the competitive factor tends to reduce the incentives offered by the principal to the agents. Conversely, process-oriented competition yields the opposite effect. The fundamental difference lies in the nature of the competition. Process-oriented competition is an endogenous mechanism; when two agents operate within the same market, interaction and competition are inherent, primarily reflecting the ability to capture resources from one another, for which the principal must compensate. Result-oriented competition, however, is an exogenous mechanism imposed by the principal to further incentivize agent efforts. For example, a company might rank regional sales managers based on performance and reward top performers, sometimes even implementing a bottom-ranking elimination system. This externally imposed competitive pressure acts as a form of incentive itself, thus explaining why the result-oriented competition factor reduces the explicit incentives offered by the principal.

**Corollary 4.** The result-oriented competition model can motivate agents to exert more efforts. The effort exerted by agent *A*_*i*_ is given by


$${\hat{a}}_{i}(t)=\frac{2k_{i}^{2}+e^{-rT}J_{i}c_{i}\rho_{i}\sigma_{i}^{2}(1+e^{-rT})}{2k_{i}^{2}+c_{i}\rho_{i}\sigma_{i}^{2}(1+e^{-rT})}\cdot \frac{k_{i}}{c_{i}}.$$

Similar to Corollary 2, Corollary 4 demonstrates that in a result-oriented competitive environment, competition incentivizes agents to exert greater efforts. Furthermore, as agents’ production efficiency, *k*_*i*_, increases, they exert more efforts. Notably, under the result-oriented competition model, agent incentives and efforts are independent of their competitors. This is because result-oriented competition is an exogenously imposed mechanism, where the agents’ value processes are relatively independent. As for which competition-incentive scheme is superior, this paper will discuss it in the following section.

## 4 Comparative static analysis

Based on the conclusions drawn in the previous sections, this section visualizes the findings using Matlab and further interprets the existing conclusions. A comparative static analysis of the two competitive systems will be conducted from perspectives such as contractual incentives, agents’ effort, principal’s utility, and the actual wage levels of the agents. Main parameter values are as follows: $\rho_{i}=\sigma_{i}=c_{i}=1,r=0.1,j_{-i}=0.5,\bar{U}=0$, $k_{1}=k_{2}=1$.

### 4.1 Comparison of contracts under two competitive modes

**Fig 2:**
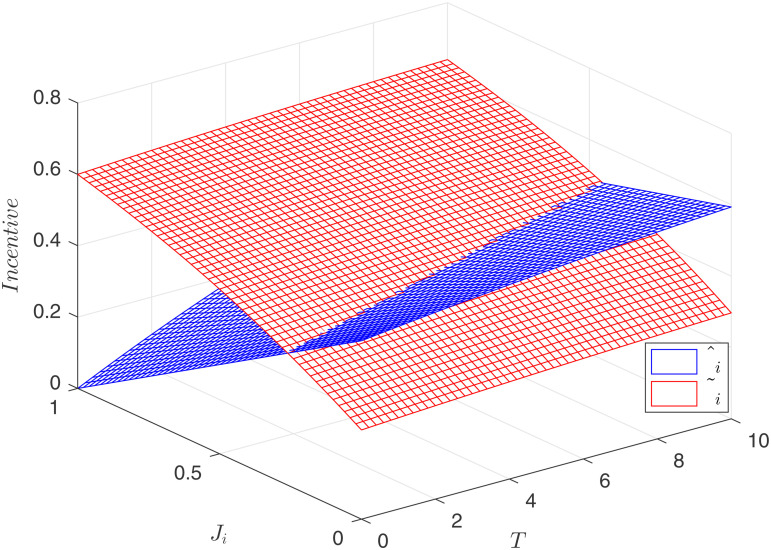
Comparison of optimal incentives.

Fig 2 illustrates the relationship between the incentive factor, contract duration *T*, and competition intensity *J*_*i*_ under both competitive mechanisms. The figure shows that both incentive factors increase with contract duration *T*, indicating that longer contract durations require more incentives from the principal. Under process-oriented competition, the incentive $\tilde{\beta }$ increases with competition intensity *J*_*i*_, demonstrating complementarity between the incentive factor and process competition, as confirmed by Corollary 1. Conversely, under result-oriented competition, the incentive factor $\hat{\beta }$ decreases as competition intensity increases, indicating substitutability, as confirmed by Corollary 3. Consequently, as shown in Fig 2, $\tilde{\beta }<\hat{\beta }$ when *J*_*i*_ is low, and $\tilde{\beta }>\hat{\beta }$ when *J*_*i*_ is high.

**Fig 3:**
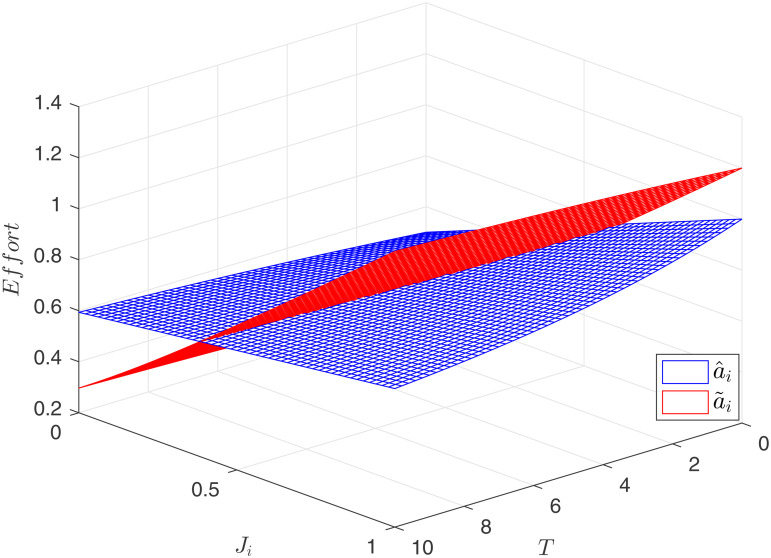
Comparison of optimal efforts.

High incentives lead to increased efforts. As depicted in Fig 3, when *J*_*i*_ is low, $\tilde{a}<\hat{a}$, and when *J*_*i*_ is high, $\tilde{a}>\hat{a}$. Higher incentives imply that the principal must allocate a larger share of the output’s returns to the agents, increasing incentive costs but also motivating greater agent efforts. The following section investigates which competitive model maximizes the principal’s returns.

Under both competitive models, effort increases with competition intensity. However, the effect of contract duration *T* differs between the two environments. In process-oriented competition, the relationship is straightforward: effort $\tilde{a}$ consistently increases with *T*. In result-oriented competition, when competition intensity is low, effort $\hat{a}$ is positively correlated with *T*. However, as competition intensifies, effort $\hat{a}$ paradoxically decreases with increasing *T*. As previously established, incentives always increase with contract duration *T*, regardless of the competitive context. Yet, in result-oriented competition, effort does not consistently follow this trend. This is because result-oriented competition resembles a gamble, where two agents effectively wager on their relative performance at time *T*. When competition is intense, the associated risks are substantial, and these risks outweigh the positive effect of incentives on agent effort, ultimately leading to a decrease in effort.

### 4.2 Comparison of profits under two competition modes

**Fig 4:**
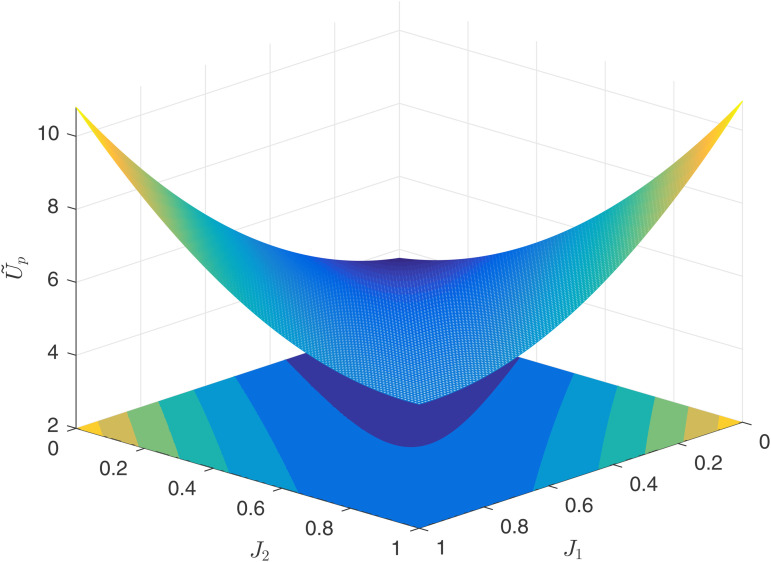
Principal’s utility ${\tilde{U}}_{P}$.

Fig 4 reflects the relationship between the principal’s utility ${\tilde{U}}_{P}$ and the competitive factors $J_{1},J_{2}$ under process competition. Since process competition is essentially a manifestation of the agent’s ability, the principal’s benefits are minimized when $J_{1}=J_{2}=0$. By observing the contour lines at the bottom, it can be seen that the principal’s utility does not reach its maximum at $J_{1}=J_{2}=1$, but rather at $J_{1}=1,J_{2}=0$ and $J_{1}=0,J_{2}=1$. Therefore, the following conclusion can be drawn.

**Numerical Conclusion 1.** Under the process-oriented competition model, when facing agents with varying levels of competitiveness in the market, the principal’s choice of a “strong+weak” combination is superior to a “strong+strong” combination, which in turn is superior to a “weak+weak” combination.

This conclusion indicates that the principle of negative assortative matching should be followed when pairing agents in terms of competitive strength. Pairing two employees with strong competitive strength can lead to ineffective competition and a waste of effort. This can be illustrated by the following matrix Table 2.

**Table 2:** The contribution of the agents to the utility of the principal.

Agent 1	Agent 2	
	Strong competitiveness	Weak competitiveness
Strong competitiveness	(3,3)	(9,1)
Weak competitiveness	(1,9)	(2,2)

Negative assortative matching is not unusual. Existing literatures has shown that when facing risk, agents follow the principle of negative assortative matching, where risk-averse individuals choose to pair with risk-takers, forming an optimal equilibrium state [23]. Additionally, when building a team of experts, to ensure diversity, it is also necessary to avoid positive assortative matching, making negative assortative matching a better choice [24]. Research has indicated that in personal asset matching, especially in marital relationships, there is also a phenomenon of negative assortative matching [25,26].

**Fig 5:**
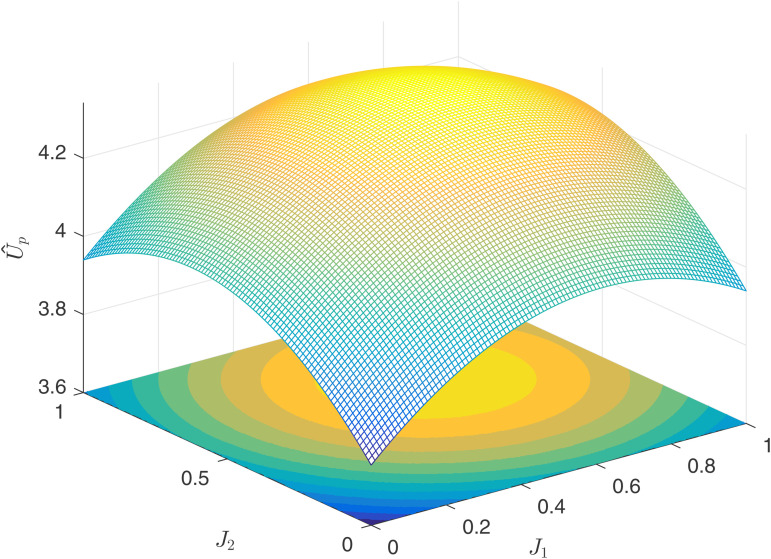
Principal’s utility ${\hat{U}}_{P}$.

Fig 5 reflects the relationship between the principal’s utility ${\hat{U}}_{P}$ and the competitive factors $J_{1},J_{2}$ under the result-oriented competition mechanism. Observations reveal that the optimal values $\left(J_{1}^{*},J_{2}^{*}\right)$ are not corner solutions. Therefore, the following conclusions can be drawn.

**Numerical Conclusion 2.** The principal can establish an result-oriented competitive mechanism for multiple agents to increase individual returns, but the intensity of competition should not be too high. Excessive competition can ultimately diminish the principal’s returns.

Result-oriented competition is an externally imposed mechanism, Corollary 3 and 4 indicate that this mechanism is effective, capable of prompting agents to exert more efforts with reduced incentives. However, as mentioned in Section 3.2, result-oriented competition can trigger additional competitive risks $\text{Var}(\Delta_{i})$. When *J*_*i*_ is very high, agents must bear significant competitive risks. Nevertheless, the principal must ensure the agents’ reservation utility $\bar{U}$, thus the costs brought about by competitive risks are ultimately borne by the principal. When the competition intensity is too high, the risk premium cost can offset the benefits brought by the establishment of the competitive mechanism.

**Fig 6:**
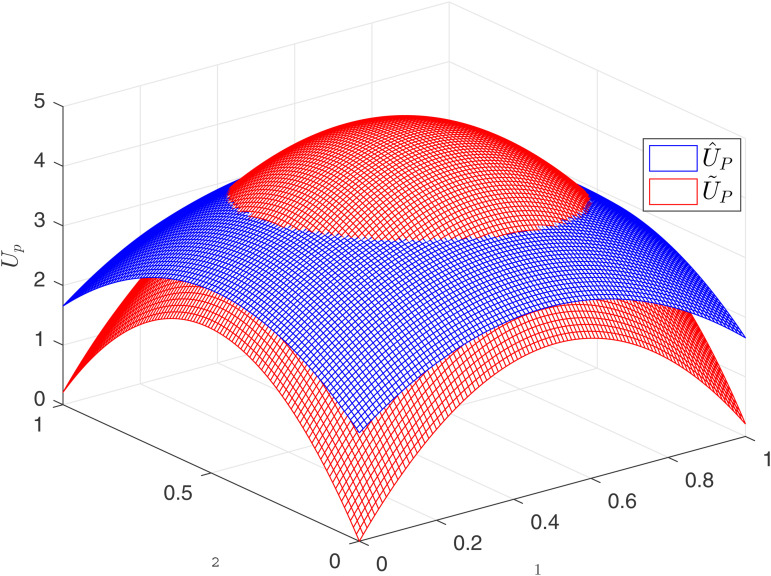
Comparison of principal utility under the same level of incentive intensity.

Based on the analysis of the impact of two types of competition on the principal’s returns, we continue to examine how the principal makes choices between the two competitive systems. In reality, the choice of competitive mechanisms does not necessarily occur simultaneously with the design of incentive mechanisms. The principal may design the incentive mechanism first and then choose to use the competitive mechanism to further enhance the agents’ effort levels and mitigate moral hazard issues. Therefore, this section discusses how the principal selects the competitive mechanism after the incentive factors have been determined. Fig 6 reflects the relationship between the principal’s returns and incentives under two competitive mechanisms. It can be observed that in the intermediate area, $\tilde{U}>\hat{U}$; while on the periphery, $\tilde{U}<\hat{U}$. Consequently, the following conclusions are drawn.

**Numerical Conclusion 3.** If high incentives or low incentives have already been implemented for one party within the team of agents, choose the result-oriented competition model; otherwise, opt for the process-oriented competition model. This conclusion can be represented by Table 3:

**Table 3:** Choosing a competitive mechanism after the incentive mechanism has been determined.

$\beta_{2}$	$\beta_{1}$		
	Low Incentives	Moderate Incentives	High Incentives
Low Incentives	Result-oriented	Result-oriented	Result-oriented
Moderate Incentives	Result-oriented	Process-oriented	Result-oriented
High Incentives	Result-oriented	Result-oriented	Result-oriented

Numerical Conclusion 3 can be explained through three scenarios.

Scenario 1: When Agent *A*_*i*_ is given low incentives. Under the process-oriented competition model, agent *A*_*i*_ will also exert little efforts. This is because, in a process-oriented competition model, the principal’s incentives are the sole motivation for the agent’s efforts. The gains from success and the losses from failure are proportional to the incentive factor. However, under the result-oriented competition model, Agent *A*_*i*_ will exert efforts due to the fear of punishment. In the result-oriented competition model, the agent’s motivation comes not only from incentives but also from the principal’s deliberate imposition of outcome comparisons, making the result-oriented mechanism superior and more effective at stimulating the agent’s efforts.

Scenario 2: When all agents are given low incentives. Since there is no collusion between the two agents, this can be interpreted as a transformation of scenario 1.

Scenario 3: When all agents receive high incentives, excessive efforts occur regardless of the competition model. This implies that the marginal benefit of effort is less than its marginal cost. As illustrated in Fig 6, under process-oriented competition, the deviation of agent effort from the optimal level is greater when excessive incentives are implemented. The fundamental reason is that the reaction function of effort to incentives is different under the two competition mechanisms. Under process competition, ${\tilde{a}}_{i}(t)=\frac{\beta_{i}(k_{i}+J_{i})}{c_{i}}$. Under outcome competition, ${\hat{a}}_{i}(t)=\frac{k_{i}\left[\beta_{i}(T)+e^{-rT}J_{i}\right]}{c_{i}}.$

**Fig 7:**
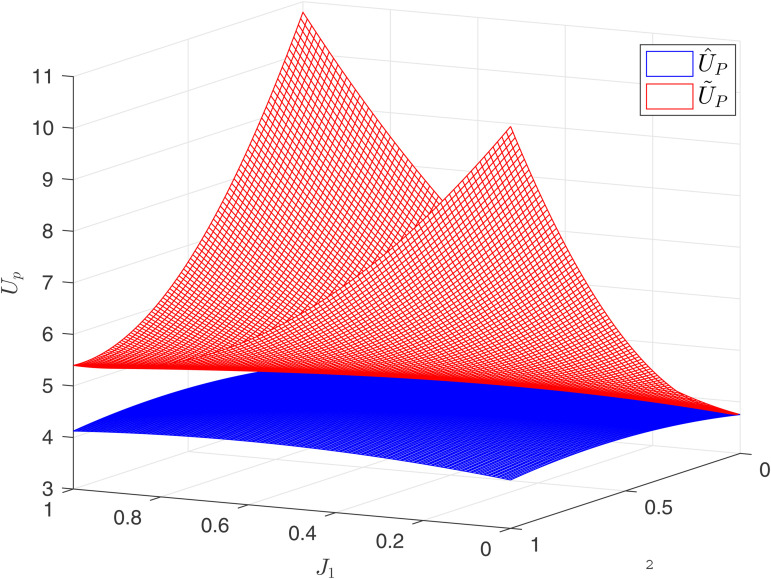
Comparison of principal utility under the same level of competition intensity.

Furthermore, consider the principal designing both competition and incentive mechanisms simultaneously. Combining Figs 4 and 5 to form Fig 7, this figure illustrates the relationship between the principal’s returns and the competitive intensities *J*_1_ and *J*_2_. It is clear that in Fig 7, process-oriented competition is superior to outcome competition. However, process-oriented competition itself is a reflection of the agents’ capabilities and cannot be imposed. Fig 7 indicates that, compared to the endogenous process-oriented competition, the exogenous result-oriented competition imposed by the principal actually yields minimal benefits.

**Numerical Conclusion 4.** The impact of result-oriented competition on the principal’s returns is not significant compared to the process-oriented competition model.

Numerical conclusion 4 indicates that the result-oriented competition model yields minimal benefits. Taking the example of agency sales, if only one retailer is assigned to each city and their annual sales results are ranked, with penalties and rewards given based on these rankings, this management method is a typical example of an result-oriented competition model. Numerical conclusion 4 suggests that if the principal can assign multiple agents to the same area and consciously establish competitive relationships during the sales process, the effect will be significantly better than an result only competition model.

The extreme case of result-oriented competition is the last-place elimination system. In recent years, it has been observed that an increasing number of companies are abolishing the last-place elimination system and the outcome-only assessment system, indicating that there is controversy over the effectiveness of this system in actual management, which corroborates Numerical Conclusion 4. In fact, the last-place elimination system is illegal in China. According to the “Minutes of the Eighth National Court Civil and Commercial Trial Work Conference (Civil Part)” published by the Supreme People’s Court of China, if an employer unilaterally terminates a labor contract during the term of the contract through “last-place elimination system” and other similar forms, the worker may request the employer to continue performing the labor contract or pay compensation on the grounds that the employer has illegally terminated the labor contract.

### 4.3 Competition intensity setting

As previously noted, the principal can only control the intensity of competition under the result-oriented competition model. Therefore, this section focuses on how to set the optimal level of competitive intensity.

In addition to reporting the optimal competitive intensity $\left(J_{1},J_{2}\right)$ for changes in production efficiency in Table 4, it is also important to provide the corresponding expected payoffs for the principal and the agents. Therefore, Table 4 also includes the expected utility of the principal and the expected payoffs of the agents for each parameter configuration $\left(E({\hat{\Pi }}_{P}),E[{\hat{\Pi }}_{A_{1}}],E[{\hat{\Pi }}_{A_{2}}]\right)$. In Table 4, the optimal competitive intensity $\left(J_{1},J_{2}\right)$ that the principal should adopt under the result-oriented competition model is given for different production efficiencies. Analyzing the relationship between production efficiency and optimal competition, the following conclusion is drawn.

**Table 4:** Competition intensity selection.

	*k*_2_ = 1	*k*_2_ = 2	*k*_2_ = 3	*k*_2_ = 4	*k*_2_ = 5
*k*_1_ = 1	(0.62,0.62)	(0.62,0.81)	(0.62,0.86)	(0.62,0.88)	(0.62,0.89)
	(4.34,0.68,0.68)	(13.52,0.68,1.16)	(29.24,0.68,1.31)	(51.34,0.68,1.36)	(79.77,0.68,1.39)
*k*_1_ = 2	(0.81,0.62)	(0.81,0.81)	(0.81,0.86)	(0.81,0.88)	(0.81,0.89)
	(13.52,1.16,0.68)	(22.70,1.16,1.16)	(38.42,1.16,1.31)	(60.52,1.16,1.36)	(88.95,1.16,1.39)
*k*_1_ = 3	(0.86,0.62)	(0.86,0.81)	(0.86,0.86)	(0.86,0.88)	(0.86,0.89)
	(29.24,1.31,0.68)	(38.42,1.31,1.16)	(54.15,1.31,1.31)	(76.24,1.31,1.36)	(104.70,1.31,1.39)
*k*_1_ = 4	(0.88,0.62)	(0.88,0.81)	(0.88,0.86)	(0.88,0.88)	(0.88,0.89)
	(51.34,1.36,0.68)	(60.52,1.36,1.16)	(76.24,1.36,1.31)	(98.34,1.36,1.36)	(126.80,1.36,1.39)
*k*_1_ = 5	(0.89,0.62)	(0.89,0.81)	(0.89,0.86)	(0.89,0.88)	(0.89,0.89)
	(79.77,1.39,0.68)	(88.95,1.39,1.16)	(104.70,1.39,1.31)	(126.80,1.39,1.36)	(155.20,1.39,1.39)

**Numerical Conclusion 5.** When facing agents with different efficiencies, the principal should set different competitive contracts, with competition intensity increasing with agent efficiency.

Numerical conclusion 5 indicates that implementing fair competition within an enterprise is not optimal. Agents with higher efficiency should be subject to more intense reward and punishment mechanisms. Taking sales as an example, if manufacturer *P* has two retailers, *A*_1_ and *A*_2_, located in different cities with almost no business overlap, to stimulate the efforts of both retailers, manufacturer *P* has established an result-oriented competitive mechanism on top of the existing revenue-sharing system, rewarding and penalizing them based on their sales volumes. However, due to the different capabilities of the sales teams, it is necessary to tailor the competition-incentive contracts with different intensities. If *A*_1_ has a higher sales efficiency, a higher competitive intensity should be set for them. This means that when *A*_1_ performs well, they will receive greater rewards than *A*_2_, and when *A*_1_ performs poorly, they will also face more severe penalties.

### 4.4 Expected salary levels under two competition models

All of the preceeding analyses are based on the perspective of the principal’s optimization. This section will analyze the impact of the two competition models on the agents’ expected salary *Y*(*T*).

The expected salary for an agent is the compensation minus the cost of effort. According to equation (1) and Proposition 1, the expected salary for Agent *A*_*i*_ under process-oriented competition is


$$\begin{array}{cc} & {\tilde{Y}}_{i}(T)\\ & =E[{\tilde{\Pi }}_{A_{i}}(T)]\\ & =\beta_{i}\left[\int_{0}^{T}e^{-rt}k_{i}a_{i}(t)dt+J_{i}\int_{0}^{T}e^{-rt}[a_{i}(t)-a_{-i}(t)]dt\right]+\int_{0}^{T}e^{-rt}\alpha_{i}(t)dt\\ & \;-\int_{0}^{T}\frac{e^{-rt}c_{i}}{2}{a_{i}}^{2}(t)dt\\ & =\bar{U}+\frac{\rho_{i}\sigma_{i}^{2}(1-e^{-2rT})}{4r}{\left(\frac{2{\left(k_{i}+J_{i}\right)}^{2}-2J_{-i}(k_{i}+J_{i})}{2{\left(k_{i}+J_{i}\right)}^{2}+\rho_{i}\sigma_{i}^{2}c_{i}(1+e^{-rT})}\right)}^{2}. \end{array}$$ (7)

Similarly, based on equation (4) and Proposition 2, the expected salary for agent *A*_*i*_ under the result-oriented competitive system can be derived.


$$\begin{array}{cc} & {\hat{Y}}_{i}(T)\\ & =E[{\hat{\Pi }}_{A_{i}}(T)]\\ & =\beta \int_{0}^{T}e^{-rt}k_{i}a_{i}(t)dt+e^{-rT}J_{i}E(\Delta_{i})+\int_{0}^{T}e^{-rt}\alpha_{i}(t)dt-\int_{0}^{T}\frac{e^{-rt}c_{i}}{2}{a_{i}}^{2}(t)dt\\ & =\bar{U}+\frac{\rho_{i}}{2}\left[{\left(\frac{2k_{i}^{2}(1-e^{-rT}J_{i})}{2k_{i}^{2}+c_{i}\rho_{i}\sigma_{i}^{2}(1+e^{-rT})}\right)}^{2}\frac{\sigma_{i}^{2}(1-e^{-2rT})}{2r}+e^{-2rT}J_{i}^{2}\frac{\left(\sigma_{1}^{2}+\sigma_{2}^{2})(1-e^{-2rT}\right)}{2r}\right]. \end{array}$$ (8)

According to equations (7) and (8), the following figures are obtained.

**Fig 8:**
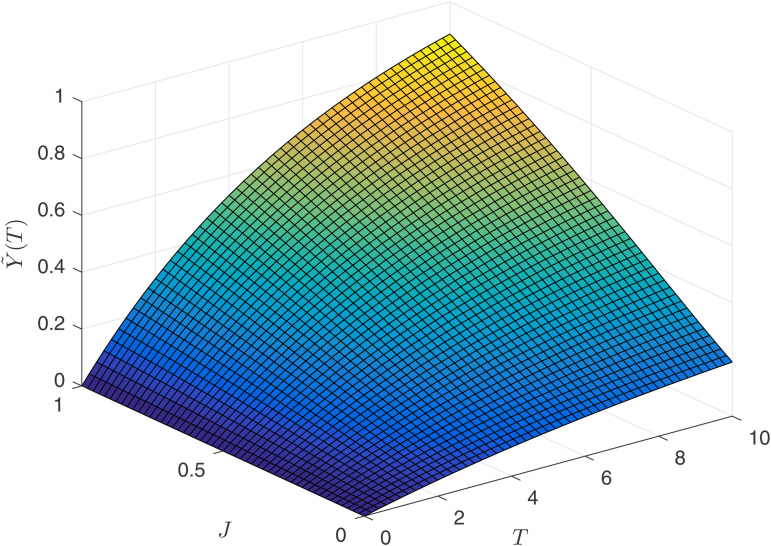
Expected returns ${\tilde{Y}}_{1}(T)$ for the agent.

**Fig 9:**
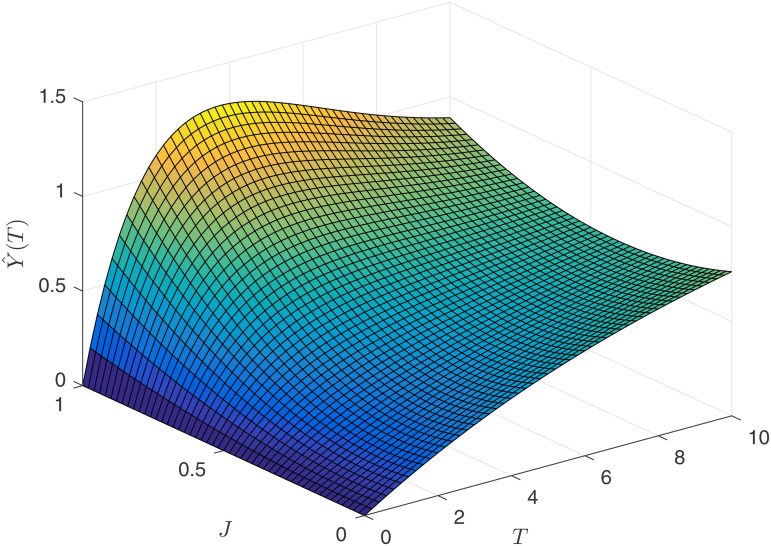
Expected returns ${\hat{Y}}_{1}(T)$ for the agent.

Fig 8 illustrates the relationship between the agent’s expected salary ${\tilde{Y}}_{1}(T)$, competitive intensity *J*_1_, and contract duration *T* in a process-oriented competitive environment. It can be observed that the agent’s expected benefits increase with the length of the contract term, and individual competitive ability also promotes their expected benefits. Fig 9 depicts the relationship between the agent’s earnings ${\hat{Y}}_{1}(T)$, competitive intensity *J*_1_, and contract duration *T* in an result-oriented competitive environment. The changes in the agent’s expected benefits are more complex. Firstly, when the competitive intensity is at an intermediate level, the agent’s expected salary level is the lowest. Secondly, when the competitive intensity is high, the agent’s expected salary level exhibits an inverse U-shaped relationship with the contract duration *T*. Therefore, if an agent places great emphasis on their own expected benefits, they would be reluctant to enter into a long-term contract with the principal when facing high-intensity competition.

**Fig 10:**
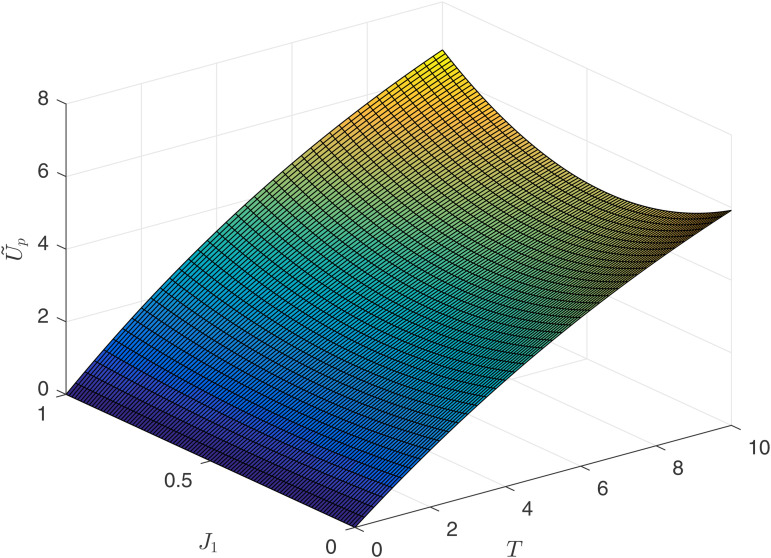
Principal’s income ${\tilde{U}}_{p}$.

**Fig 11:**
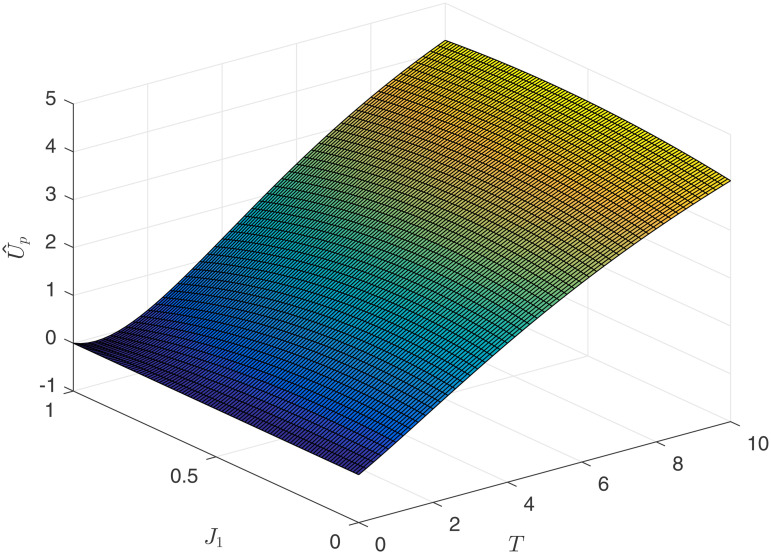
Principal’s income ${\hat{U}}_{p}$.

Figs 10 and 11 reflect the relationship between the principal’s earnings ${\tilde{U}}_{p}$ and ${\hat{U}}_{p}$, competitive intensity *J*_1_, and contract duration *T*. By comparing Figs 8, 9 with Figs 10 and 11, the following numerical conclusion is drawn.

**Numerical Conclusion 6.** In the result-oriented competition model, setting a competition factor that is too high by the principal carries the risk of talent loss.

Upon observing Figs 10 and 11, it is found that the principal’s earnings are increasing with respect to time. Therefore, regardless of the type of competition model, the principal would like to enter into long-term contracts with the agents. However, if the principal chooses an result-oriented competition model and sets the competition intensity very high, it may lead to the consequence of not being able to retain talent, as reflected by the conclusions from Fig 9. If the principal chooses a process-oriented competition model, there is no need to worry about the length of the contract. Observing Figs 8 and 10, under the process competition model, the earnings of both the principal and the agent are positively correlated with time *T*.

## 5 Conclusion

Due to the potential for mutual free-riding among multiple agents, principals often implement competitive mechanisms. This paper analyzes and compares the effects of process-oriented and result-oriented competitive mechanisms on agent behaviors and principal returns. Our comparative analysis yields the following conclusions.

First, under process-oriented competition, where multiple agents operate within the same market, increased effort by one agent can negatively impact others. The principal should provide greater incentives to agents with stronger competitive abilities. When pairing agents with differing competitive strengths, a “strong-weak” combination is preferable to a “strong-strong” combination, which, in turn, is preferable to a “weak-weak” combination, mitigating the risk of mutually detrimental outcomes.

Second, under result-oriented competition, agents operate relatively independently, and the principal rewards or penalizes them based solely on final sales or outputs. The optimal contract’s competition intensity is positively correlated with agent effort efficiency, rendering uniform competition across agents with heterogeneous efficiencies suboptimal. Furthermore, incentives and competition act as substitutes; the principal can reduce incentive costs by intensifying competition. However, excessive competition can lead to increased competitive risk and overexertion, thus necessitating careful calibration of competition intensity.

Finally, compared to contracts without competition, both competitive models effectively incentivize greater agent efforts. Moreover, under these competitive models, the incentives offered by the principal increase with contract duration, indirectly supporting the importance of incentives for talent retention. In practice, these two competitive mechanisms correspond to distinct management styles. When managing multiple agents, the principal can either assign multiple agents to the same market to foster internal competition or assign each agent to an independent market and then rank them based on sales performance, imposing external competition. Our findings indicate that both systems are effective, but process-oriented competition yields greater utility gains for the principal compared to result-oriented competition.

Applying these findings to supply chain management, we derive the following practical implications and managerial insights: 1) Implementing uniform competition within the supply chain is suboptimal; retailers with higher profit efficiency should be subject to stronger reward and punishment mechanisms. 2) Managers should calibrate competition intensity based on retailers’ actual profit efficiency, recognizing the positive correlation between the two; excessively intensifying competition can ultimately diminish the manufacturer’s profits. 3) Introducing multiple retailers in the same area can stimulate competition, but careful matching is crucial; a “strong-weak” combination is generally preferable to a “strong-strong” combination, which is, in turn, preferable to a “weak-weak” combination. 4) Introducing multiple retailers in the same market to trigger internal competition is better than ranking retailers based on sales performance and then implementing rewards and punishments, which is an external competition model.

This study has some limitations that suggest directions for future research. While this paper examined the two competitive incentive models independently, it does not consider the effects of combining them. Exploring the mixed use of these two mechanisms is a valuable avenue for future research.

## Supporting information

S1 Appendix Proofs of the main propositions. This file includes the proofs of Proposition 1 and Proposition 2.
(PDF)
