# Supplementary material for: Design, comparison and selection of two competition incentive mechanisms
Source: PLoS One. 2026 Apr 1;21(4):e0345083. doi: 10.1371/journal.pone.0345083 (PMC13043063; doi:10.1371/journal.pone.0345083)
Supplement: S1 Appendix — This file includes the proofs of Proposition 1 and Proposition 2. (PDF) [file pone.0345083.s001.pdf]

Appendix: Proofs of the Main Propositions

Appendix A. Proof of Proposition 1

According to the incentive compatibility condition, the first-order condition for agent A_i to optimize their own utility is

$$\beta_i e^{-rt} k_i + \beta_i e^{-rt} J_i - e^{-rt} c_i a_i(t) = 0.$$

Thus, we have

$$a_i^*(t) = \frac{\beta_i(k_i + J_i)}{c_i}.$$

From this, we can calculate the following results

The effort cost for agent A_i is

$$\int_0^T \frac{e^{-rt} c_i}{2} a_i^2(t)dt = \frac{\beta_i^2 (k_i + J_i)^2 (1 - e^{-rT})}{2rc_i}.$$

The total output for agent A_i is

$$\int_0^T e^{-rt} k_i a_i(t)dt = \frac{\beta_i k_i (k_i + J_i)(1 - e^{-rT})}{rc_i}.$$

The competitive revenue for agent A_i is

$$\int_0^T J_i e^{-rt}[a_i(t) - a_{-i}(t)]dt = \frac{J_i(1 - e^{-rT})}{r}\left[\frac{\beta_i(k_i + J_i)}{c_i} - \frac{\beta_{-i}(k_{-i} + J_{-i})}{c_{-i}}\right].$$

Substituting the participation constraint into equation (3), we get

$$\begin{aligned}\tilde{\Pi}_P(T) &= \sum_i \int_0^T e^{-rt} k_i a_i(t)dt + J_i \int_0^T e^{-rt}[a_i(t) - a_{-i}(t)]dt - \bar{U}_i - \tfrac{\rho_i}{2}\beta_i^2\sigma_i^2 \tfrac{(1-e^{-2rT})}{2r} \\ &= \sum_i \tfrac{\beta_i k_i (k_i+J_i)(1-e^{-rT})}{rc_i} + \tfrac{J_i(1-e^{-rT})}{r}\left[\tfrac{\beta_i(k_i+J_i)}{c_i} - \tfrac{\beta_{-i}(k_{-i}+J_{-i})}{c_{-i}}\right] \\ &- \sum_i \tfrac{\beta_i^2(k_i+J_i)^2(1-e^{-rT})}{2rc_i} + \bar{U}_i + \tfrac{\rho_i}{2}\beta_i^2\sigma_i^2 \tfrac{(1-e^{-2rT})}{2r}.\end{aligned}$$

The principal optimizes their own utility by designing the incentive mechanism, i.e., setting $\frac{\partial \tilde{\Pi}_P(T)}{\partial \beta_i} = 0$, we get

$$k_i(k_i + J_i) + J_i(k_i + J_i) - J_{-i}(k_i + J_i) - \beta_i(k_i + J_i)^2 - \frac{c_i \rho_i \beta_i \sigma_i^2 (1 - e^{-rT})}{2} = 0.$$

Appendix B. Proof of Proposition 2

According to the incentive compatibility constraint, the agent aims to maximize their own utility. Similar to the proof of Proposition 1, from equation (5) we have

$$a^*(t) = \frac{k_i[\beta_i(T) + e^{-rT}J_i]}{c_i}.$$

From this, we can calculate the following

The total output for agent A_i is

$$\int_0^T e^{-rt}k_i a_i(t)dt = \frac{k_i^2[\beta_i(T) + e^{-rT}J_i](1-e^{-rT})}{rc_i}.$$

The effort cost for agent A_i is

$$\int_0^T \frac{e^{-rt}c_i}{2}a_i^2(t)dt = \frac{k_i^2[\beta_i(T) + e^{-rT}J_i]^2(1-e^{-rT})}{2rc_i}.$$

Substituting the participation constraint into equation (6), we get

$$\begin{aligned}
&\hat{\Pi}_P(T) \\
&= \sum_i \int_0^T e^{-rt}k_i a_i(t)dt - \int_0^T \tfrac{e^{-rt}c_i}{2}{a_i}^2(t)dt - \tfrac{\rho_i}{4r}[\beta_i^2(T)\sigma_i^2(1-e^{-2rT}) \\
&\quad + e^{-2rT}J_i^2(\sigma_1^2+\sigma_2^2)(1-e^{-2rT})] \\
&= \sum_i \tfrac{k_i^2[\beta_i(T)+e^{-rT}J_i](1-e^{-rT})}{rc_i} - \tfrac{k_i^2[\beta_i(T)+e^{-rT}J_i]^2(1-e^{-rT})}{2rc_i} \\
&\quad - \sum_i \tfrac{\rho_i}{4r}[\beta_i^2(T)\sigma_i^2(1-e^{-2rT}) + e^{-2rT}J_i^2(\sigma_1^2+\sigma_2^2)(1-e^{-2rT})].
\end{aligned}$$

The principal optimizes their own utility by setting up the incentive mechanism, i.e., $\frac{\partial\hat{\Pi}_P(T)}{\partial\beta_i} = 0$, thus we can solve for

$$\hat{\beta}_i(T) = \frac{2k_i^2(1-e^{-rT}J_i)}{2k_i^2 + c_i\rho_i\sigma_i^2(1+e^{-rT})}.$$
